# Comparing the *In Vitro* Stiffness of Straight-DCP, Wave-DCP, and LCP Bone Plates for Femoral Osteosynthesis

**DOI:** 10.1155/2013/308753

**Published:** 2013-02-26

**Authors:** José Ricardo Lenzi Mariolani, William Dias Belangero

**Affiliations:** Laboratory of Biomaterials in Orthopedics, School of Medical Sciences, University of Campinas (UNICAMP), 126 Rua Tessália Vieira de Camargo, 13083-887 Campinas, SP, Brazil

## Abstract

The objective of this study was to compare the Locking Compression Plate (LCP) with the more cost-effective straight-dynamic compression plate (DCP) and wave-DCPs by testing *in vitro* the effects of plate stiffness on different types of diaphyseal femur fractures (A, B, and C, according to AO classification). The bending structural stiffness of each plate was obtained from four-point bending tests according to ASTM F382-99(2008). The plate systems were tested by applying compression/bending in different osteosynthesis simulation models using wooden rods to simulate the fractured bone fragments. Kruskal-Wallis test showed no significant difference in the bending structural stiffness between the three plate models. Rank-transformed two-way ANOVA showed significant influence of plate type, fracture type, and interaction plate versus fracture on the stiffness of the montages. The straight-DCP produced the most stable model for types B and C fractures, which makes its use advantageous for complex nonosteoporotic fractures that require minimizing focal mobility, whereas no difference was found for type A fracture. Our results indicated that DCPs, in straight or wave form, can provide adequate biomechanical properties for fixing diaphyseal femoral fractures in cases where more modern osteosynthesis systems are cost restrictive.

## 1. Introduction

The current standard of care for femoral fractures is the intramedullary nail, but there are situations in which the use of plates is indicated, like narrow medullary cavity, bone deformities, and open growth plate. Metal plates have been used for the fixation of fractures since the end of the 19th century. The DCP, designed with oblong holes to provide interfragmentary compression when tightening the screws, was introduced in 1969 [1]. At that time, researchers believed that “absolute” stability was the best method for treating long bone diaphyseal fractures, but a high rate of nonunions and postoperative infections questioned this concept only a few decades later [2–4]. As opposed to the wide surgical approach required by this technique, it has been suggested that less manipulation of the bone fragments would result in faster bone healing by preserving the fracture site vasculature [5]. Consequently, osteosynthesis techniques were modified [2, 4, 6–8] to minimize damage to the soft tissue and periosteum in order to preserve the fracture vascularization [2, 7]. The authoritative work of Heitemeyer et al. [9] demonstrated that this new protocol improved complex fracture healing of the femur diaphysis.

Appropriate stress distribution is fundamental to proper bone remodeling during fracture healing. Within a certain range of values, compressive stresses drive bone growth, whereas tensile stresses favor connective tissue or fibrocartilage formation [10]. Fracture and plate geometries affect the stress distribution and stiffness of the bone/plate system and must be taken into account when considering an osteosynthesis repair. Blatter et al. [11] studied the influence of the medial cortex on the stress distribution within the bone/plate system. They found that fracture sites were experiencing high stresses and plate fatigue failure in the absence of medial support. These findings led to the creation of the wave-Dynamic Compression Plate (DCP). By bending a straight-DCP in a controlled manner (forming the “wave”), a gap is created between the plate and the bone. The wave plate shifts the neutral axis toward the lateral cortical region, so that the bone is in compression, while the plate is in tension [11, 12]. Ring et al. [13] used the wave plate and reported bone consolidation in 41 of 42 patients with pseudoarthrosis of the femur diaphysis.

The Locking Compression Plate (LCP) is a more recent improvement to the straight-DCP technology that shares similar characteristics and mechanical performance to external fixators. In this system, the fixation screws are locked in the plate [14–16]. The LCP design minimizes contact with the bone, which reduces the damage to the periosteum and the resulting bone necrosis as well. The introduction of the LCP made the DCP plate obsolete, although the latter continues to be used in developing countries because of its lower cost (about 10%–20% of the LCP cost) [17].

Currently, there are no studies comparing directly the effectiveness of these three plate systems within the same model. To assess whether patients receiving the DCP plate are receiving adequate fracture care, the stiffness of each plate design was tested in simulated models of types A, B, and C diaphyseal femoral fractures (AO classification) [18].

## 2. Materials and Methods

### 2.1. Static Bending Test of the Plates

**Figure 1:**
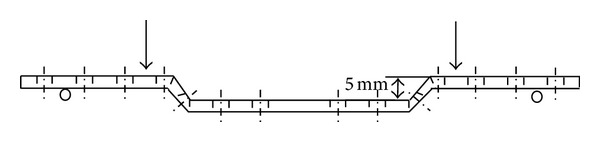
Position of support and loading points (wave-DCP).

Four-point static bending tests were conducted on 14-hole 4.5 mm wide straight- and wave-DCPs (Synthes number 226.140) and LCPs (Synthes number 226.641), according to ASTM F382-99(2008) (Standard Specification and Test Method for Metallic Bone Plates) [19]. The wave plates were shaped from straight plates as described by Blatter et al. [11]. Briefly, the plate was bent at the third and fourth screw holes on either side of the plate center to form a 5 mm high wave (Figure 1). The tests were conducted in an EMIC (Curitiba, Brazil) DL2000 testing machine at a speed of 2.0 mm/sec. A special apparatus was used to support and load the plates. According to ASTM F382-99(2008), the loading rollers should be positioned so that two plate screw holes would be located between them, while the support rollers should be placed two screw holes away from the loading rollers. For testing the wave-DCP, however, the distance between the two loading rollers had to be increased to eight screw holes to encompass the whole wave between them, while the support rollers were still positioned two screw holes away from the loading rollers (Figure 1). The straight-DCPs and LCP were tested under the same conditions as the wave-DCPs, because testing results are only directly comparable to each other when using the same loading/support roller locations [19]. According to ASTM F382-99(2008), the plate bending structural stiffness EI_*e*_ can be calculated according to (1):

(1) $$\begin{array}{c} \text{E}{\text{I}}_{e}=\frac{\left(2h+3a\right)Kh^{2}}{12}, \end{array}$$

where: *K* is the bending stiffness (slope of the linear region of the load versus load-point displacement curve); *h* is the distance between the loading rollers; *a* is the distance between a loading roller and an adjacent support roller.

Four specimens of straight-DCPs (*n* = 4), four of LCPs (*n* = 4) and seven of wave-DCPs (*n* = 7) were tested. The average stiffness and standard deviation were calculated for each plate model, and the results were compared by using Kruskal-Wallis test with a significance level of 5%. The analysis was performed by the SAS software (SAS Institute Inc., Cary, NC, USA).

### 2.2. Static Compression Test of the Montages Simulating Fractures

The test specimens were divided into three simulation groups, corresponding to types A, B, and C diaphyseal femur fractures (AO classification) [18]. Each plate type was tested in all three fracture groups. Test specimens consisted of two dense wooden rods (*Pouteria pachycarpa *Pires), 200 mm long and 26 mm in diameter, attached with a different type of osteosynthesis plate. The model used for the static compression tests has been used previously to simulate the physiological axes of the femur to approximate physiological loading conditions in experimental compression tests [20–22]. Wood has been a material of choice for such kind of tests [23–25] and was chosen, because it presents more uniform mechanical properties than human cadaveric bone and serves as a good anchor for the fixation screws [22]. Although the wooden rods do not mimic the real bone, they were used because the interest was in the relative stiffness among the montages, not in the absolute stiffness.

**Figure 2:**
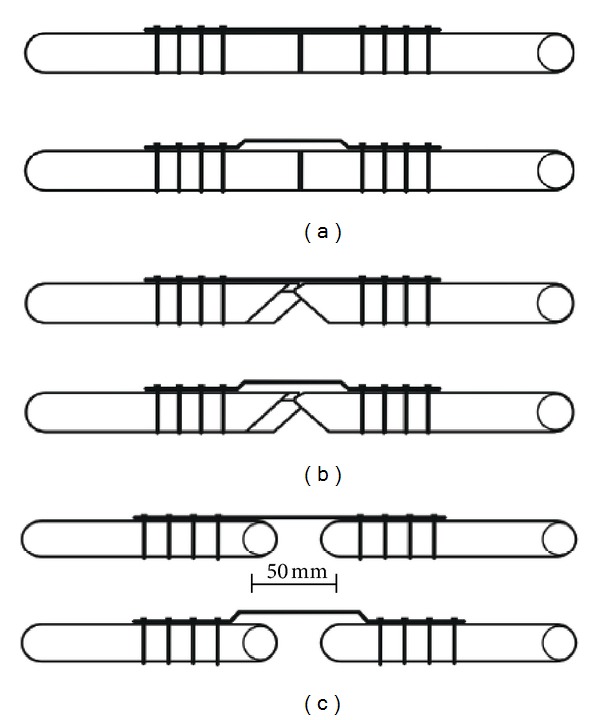
Models simulating fractures employed on the static compression tests. (a) Type A fracture; (b) type B fracture; (c) type C fracture.

**Figure 3:**
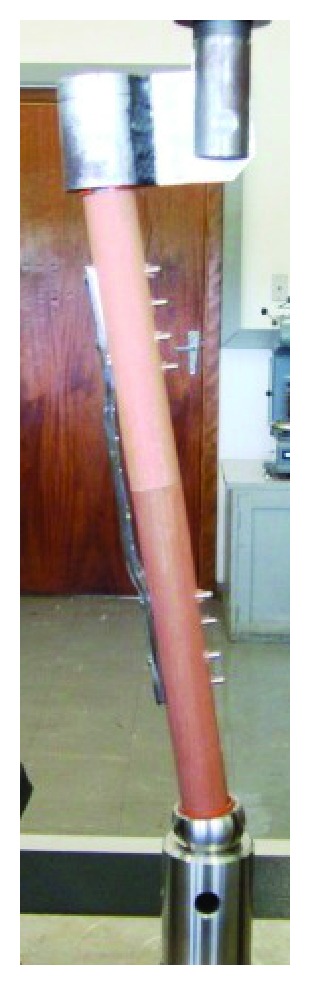
Apparatus for applying concomitantly compression and flexion to the specimens. Angle between the load application line and the specimen centerline: 9°.

Straight- and wave-DCPs (Synthes number. 226.140) were affixed to the rods using cortical screws (Synthes number. 214.034), whereas the LCPs were affixed using locking screws (Synthes number. 212.209). The first four holes relative to the plate end were used to anchor the osteosynthesis plates to the test specimens (Figure 2). All screws were tightened to 5 N*·*m torque. The fracture type was defined by the shape of the wooden rods and the distance between them. Type A fractures were simulated by attaching the two rods without any gap between them (Figure 2(a)). For type B fractures, the rods were obliquely sectioned at each end (75% of diameter) to simulate medial cortical bone loss, and the remaining areas were attached close together (Figure 2(b)). Type C fractures were simulated by creating a 50 mm gap between the two rods (Figure 2(c)). Samples using the wave plates had a slight *valgus *alignment due to the plate shape. The tests were conducted in an MTS (Eden Prairie, MN) Sintech 5G testing machine. The test specimens were externally positioned using an apparatus to reproduce physiologically relevant force axes within the sagittal-only loading condition in the MTS (Figure 3). The apparatus allowed for concomitant application of compression and flexion forces on the test specimens, similar to human femoral loading. To simulate the knee joint, a 34 mm diameter semisphere was attached to the distal end of each specimen.

Before beginning the test, a preload of 5 N was applied for system accommodation. The test was conducted at a displacement rate of 5 mm/sec, and the load was applied until there was an evident plate deformation or a fracture of the wooden rod. The specimen stiffness was calculated from the slope of the linear region of the load versus displacement curve.

Six specimens of each plate were tested within the types A and B fracture groups (*n* = 6), and five specimens of each plate were tested within the type C fracture group (*n* = 5). Two-way Analysis of Variance (ANOVA) was used to compare the stiffness of the montages considering both fracture type and plate model. A rank transformation was applied due to the small number of samples within each group. Pairwise comparison was carried out by using the Tukey test. The tests were conducted with a significance level of 5%. The analysis was performed by the SAS software (SAS Institute Inc., Cary, NC, USA).

## 3. Results

**Figure 4:**
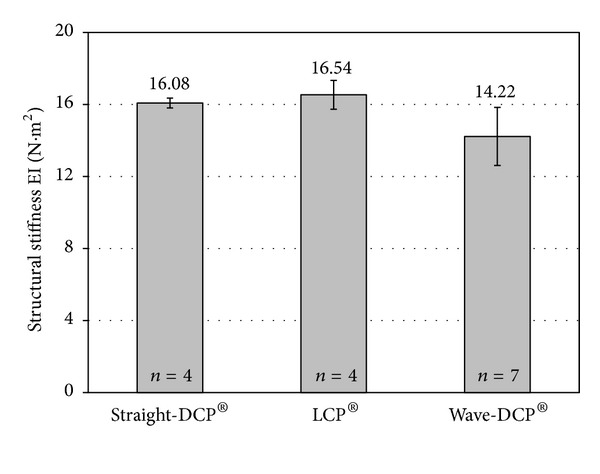
Average structural stiffnesses of the plates obtained from the static bending test. Error bars indicate standard deviations.

**Figure 5:**
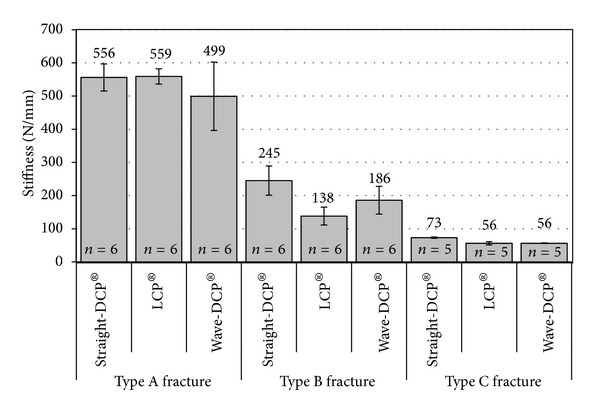
Comparison between the average stiffness obtained for each type of plate and fracture. Error bars indicate standard deviations.

Figure 4 compares the average structural stiffness of the straight-DCPs, wave-DCPs and LCPs. The Kruskal-Wallis test showed no significant difference between the three plate models (*P* = 0.0765).  Figure 5 shows the average values of stiffness obtained for the different montages simulating fractures. Rank-transformed two-way ANOVA showed significant influence of plate type (*P* = 0.0002), fracture type (*P* < 0.0001), and interaction plate versus fracture (*P* = 0.0260) on the stiffness of the montages.  Table 1 shows the Tukey test results for pairwise comparison of the fracture types according to plate type, and Table 2 shows the pairwise comparison of the plate types according to fracture type.

## 4. Discussion

The structural stiffness measured by ASTM F382-99(2008) is the most appropriate parameter for comparing the bending stiffness of different bone plates. Our results showed no significant difference between the structural stiffness of the plates (*P* = 0.0765), although the straight-DCPs and the LCPs appeared to be, in average, stiffer than the wave-DCP (Figure 4). This lack of significance could be attributed mainly to the fact that the wave-DCP shaping process was done by hand, which could have led to an important dimensional variability within the measured samples and could have biased the statistical analysis. The dimensional variation inherent to the manufacturing process of the plates is another—less important—factor of influence. This statistical dispersion would obviously not exist on a theoretical calculation of the stiffnesses, based on the nominal dimensions of the plates. In such case, the calculated values should approximate the average stiffnesses obtained from the bending tests, and the wave-DCP would be the less stiff. But the experimental results reflected the real situation in surgical practice, where the differences in stiffness between the plates may become undistinguishable due to the statistical variability.

Table 1 and Figure 5 show that the fracture type exerts the most influence on the stiffness of the models regardless of the plate type (*P* < 0.0001). Type A fracture models exhibited average stiffness two to ten times higher than that of either type B or type C. Within each fracture group, the variation of stiffness among the models was considerably smaller than the variation between fracture types. No significant differences could be found between the plates for type A fractures (*P* = 0.3456, Table 2). The straight-DCP produced the greatest stiffness for fractures types B and C. The standard deviation bars in Figure 5 show that the contact between the wooden rods (types A and B fractures) contributes to increased variation of the results, since these two fracture types exhibited greater statistical dispersion. The straight-DCP plate produced significantly stiffer type B fracture models than LCP (*P* = 0.0007, Table 2), but no significant difference was found between straight-DCP and wave-DCP and between LCPs and wave-DCPs Straight-DCPs produced also the stiffest montages for type C fracture (*P* = 0.0011, Table 2).

The stiffness of an osteosynthesis used to be considered important, because it determines the primary stability. However, a highly stiff system may not be advantageous. According to Claes and Heigele [10], intramembranous ossification occurs at strains under 5%, whereas endochondral ossification occurs at strains between 5% and 15%. Strains higher than 15% produce connective tissue or fibrocartilage. Perren [5], citing Hente et al., remarked that an absence of strain would prevent callus formation, whereas very little strain would induce it; strain up to 2% would be tolerated by lamellar bone tissue and up to 10% by three-dimensional woven bone. Strains between 10% and 30% would induce bone resorption. Moreover, under these higher strains, the gap distance between bone fragments would increase because of osteoclast-mediated resorption of the fracture ends until the strain on the repair tissue decreased enough to allow bone formation. Increased stiffness would lower the strain, but it would still be large enough to inhibit direct ossification. These strain-mediated changes could slow resorption of the fracture ends and callus formation, thereby increasing the risk of implant fatigue. Thus, relative stability is more advantageous than absolute stability for an osteosynthesis in a highly stressed lower limb fracture.

In our study, no statistically significant differences were found between the plates for type A fractures, which is in agreement with the conclusion of Karnezis [26], who compared the rigidity characteristics of bridging and wave plating. In spite of this, clinical results obtained by Angelini et al. [17] (union rate of 95%, average time of consolidation of 12.8 weeks) suggest that wave-DCPs have a potential advantage in this fracture type and further support the use of these plates for pseudoarthrosis of the femoral shaft. The tendency of the wave-DCP in producing less stiff osteosyntheses (despite the lack of statistical significance), the preservation of the periosteum provided by the wave shape, and the fact that the wave-DCP design subjects most of the fracture focus to compressive stress, while the straight-DCP puts a substantial part of the focus in tensile stress [11] could have contributed to the good results obtained by Angelini et al. [17]. Although, according to Blatter et al. [11], the wave-DCP is initially subjected to higher stresses, in the cited study [17] it probably could have relieved plate stress faster by allowing faster callus formation and bone consolidation and could have protected it against fatigue failure. If callus formation is delayed, the plate may eventually suffer fatigue failure, but the wave plate would still last longer than a straight plate of the same dimensions [17].

Factors like heterogeneity and anisotropy of the fragment's material, misalignment between the fragments and hand-shaping of the wave plates are unavoidable in surgical practice. Accounting for these factors, it is possible that the wave-DCP may not always produce better results than the straight-DCP, as found by Angelini et al. [17].

The straight-DCP exhibited the highest stiffness in the compression testing of the type C fracture models. In opposition to simple fractures, higher primary stability (or, in this case, lower instability) may be advantageous for complex fractures. In this case, the use of a wave plate would not be advantageous.

For type B fractures, the straight-DCP provided also the stiffest fixation, but surprisingly, the LCP plate produced the less stiff montages. The deformation pattern differed from that of types A and C fractures, because the lateral contact between the fragments acted as a pivoting point. This most likely occurs in clinical practice, but it is very difficult to generalize across type B fractures due to their almost infinite geometric variability, which can influence the performance of different implants.

In our model, the three types of plates exhibited similar results for type A fracture. The more modern LCP should be chosen if there is no cost restriction, then it minimizes the injury to the tissue. The wave-DCP, which also produces less tissue damage and exhibited good clinical results [17], could be used if cost is an important issue. The straight-DCP provided the highest primary stability for fractures types B and C. This higher stability suggests that, for healthy bones, the straight-DCP is the most suitable for fixing complex type C fractures that need to minimize mobility in the fracture focus. These considerations made here are valid only for healthy bones where the cortical screws can be tightened enough to prevent relative slippage between the screw head and the plate. Otherwise, the low cost advantage could be eliminated by the fail of the treatment. For osteosynthesis of osteoporotic bone, when it is not possible to apply the required 5 N·mm torque employed in this study [27], the LCP with locking screws is the most appropriate choice [28].

## 5. Conclusions

From the point of view of the stiffness, the DCP can still be safely used for the osteosynthesis the of healthy (non-osteoporotic) bones, either as wave plate for simple fractures or as straight plate for complex fractures, in situations where the high cost of the LCP is a problem for the patient or the local welfare system.

## Figures and Tables

**Table 1:** Pairwise comparison between fracture types for each plate type (Tukey's test).

Plate	*P*	Significant differences between the following fractures
Straight-DCP	<0.0001	A versus B; A versus C; B versus C
Wave-DCP	<0.0001	A versus B; A versus C; B versus C
LCP	<0.0001	A versus B; A versus C; B versus C

**Table 2:** Pairwise comparison between plates for each fracture type (Tukey's test).

Fracture	*P*	Significant differences between the plates
A	0.3456	None
B	0.0007	Straight-DCP versus LCP
C	0.0011	Straight-DCP versus LCP; Straight-DCP versus wave-DCP
